# Regional Anatomy in Its Relation to Medicine and Surgery

**Published:** 1892-02

**Authors:** 


### Book Reviews.

REGIONAL ANATOMY IN ITS RELATION TO MEDICINE AND SURGERY. By GEORGE MCCLELLAN, M. D., Lecturer on Descriptive and Regional Anatomy at the Pennsylvania School of Anatomy ; Professor of Anatomy at the Pennsylvania Academy of the Fine Arts ; Member of the Association of American Anatomists, Academy of Natural Sciences, Academy of Surgery, College of Physicians of Philadelphia, etc. Illustrated from photographs taken by the author, of his own dissections, expressly designed and prepared for this work, and colored by him after nature. "L'anatomie n'est pas telle qu'on l'enseigne dans les ecoles."—Bichat. In two quarto volumes. Vol. I. Philadelphia : J. B. Lippincott Company. 1891.

No one can read Prof. McClellan's Regional Anatomy without having two conclusions impressively forced upon him, viz., that the author is a most excellent and practical anatomist, and that he has little or no sympathy with the methods of imparting anatomy to students ordinarily used by teachers of this branch of science. There can no longer be any doubt that regional anatomy, the anatomy of the different regions of the body considered in the relation of their various parts to one another, is the only scientific, and therefore the only practical and profitable way for the teacher to present, and the student to study, this fundamental science. We were very glad, therefore, when we learned that the author was doing for American practitioners and students what Treves and Tillaux, for example, have done so well in the old world. That he has succeeded, no one can doubt who reads this magnificent work.

Vol. I. is concerned with the head and upper regions of the body, including thorax and upper extremity. The head alone is given sixty pages, and is quite typical of the thoroughness which characterizes the work. Beginning with the surface landmarks of the skull, each portion of this region is described as successive dissections have brought the various planes into view. Wherever an application of anatomical knowledge can be made to medicine or surgery, the author has made it. Especially interesting and valuable in this connection are those pages devoted to the topography of the brain. No part of the work shows the happy combination of anatomy and surgery in the author better than his remarks upon localization of lesions from the operative standpoint. He says, "That the results of operative interference with the brain-structure should so often verify diagnosis, is cause for wonder, when one considers the uncertain features of the anatomy of localization. Repeated examinations of the relations of the fissures to carefully mapped-out points, after removal of a disc of bone on the heads of many cadavers, have shown the author the fallacy of depending solely upon measurement, and the importance of making the artificial opening in the skull large enough to enable the operator to see the parts exposed." We think this quite coincides with the experience of surgeons who have had to do with this class of cases.

The ear, eye, nose, face, mouth, larynx, and neck are in turn considered, with their immediate surrounding regions, and wherever a plate serves to illustrate the text, it is found. Especially handsome and clear are the plates showing the various structures of the neck, and, together with the excellent text descriptive of this important region, this portion of the work will prove of decided value to the reader.

The chapter upon the Thorax is also very complete, and contains many practical suggestions whereby the anatomy of this region may be turned to clinical use, and the same remark applies with equal force to the treatment of the upper extremity, which forms the closing chapters of this volume.

The dry and uninteresting descriptions, from the medical student's standpoint, which so abound in the average text-book that is put into his hands, are nowhere found in this book. The style is clear, easy, and simple, and such refinements as tend only to confuse, the author has judiciously omitted. While it is often apparent that the author has read and extracted the meat from the standard writers upon practical anatomy, still the effect of the book, as a whole, is that it is the result of large information derived from actual dissection and mature clinical experience, and these, after all, are the best books.

Beside the method of the work, the next most important feature is found in the character of the illustrations. These aim to be exact representations of the dissections of the author, and to this end the camera has been employed. The parts have been first photographed, and from these the plates were made on stone, the object in employing photography being to reproduce such exact representations of the dissections as would illustrate and verify the descriptions given in the text. Admirable as is the result in general, there are a few plates in the book which are not distinct, and in these instances it seems to us that a diagram would serve a better purpose than the truer but less clear photographic picture. Wherever many different structures are represented in a small space, as for instance the views of the brain, the general effect is not good, and one must strain his eyes to differentiate the various parts. Not so, however, the region of the neck, where, for the most parts, the picture is a model of beauty and clearness. Looked at as a whole, the book is a great credit to the author and a most handsome and elaborate specimen of book-making. We always expect the very best from the house of Messrs. Lippincott, but they have, in this instance, done more than fulfil our expectations. The work is worth the price asked, and deserves a widespread circulation among practitioners and students. J. P.

